# Doxycycline Sclerotherapy of a Cervical Cystic Hygroma: A Caribbean Institution Experience

**DOI:** 10.1155/2020/9187205

**Published:** 2020-09-15

**Authors:** Nicholas Figaro, Robbie Rampersad, Solaiman Juman

**Affiliations:** ^1^Department of Clinical Surgical Sciences, University of the West Indies, Eric Williams Medical Sciences Complex, Champs Fleur, Trinidad and Tobago; ^2^Department of Radiology, University of the West Indies, Eric Williams Medical Sciences Complex, Champs Fleur, Trinidad and Tobago

## Abstract

Cervical cystic lymphangiomas are rare benign tumors that pose a formidable challenge to surgeons confronted with managing this uncommon entity. Due to the intimacy with which these cystic lesions blend with critical cervical structures, a considerable number of patients who undergo surgical management are often plagued with recurrence and morbidity. As a result, doxycycline sclerotherapy has become an attractive, safe, and effective alternative as a primary treatment modality in a select group of pediatric patients. This case report presents an 18-month-old patient with a large cervical cystic hygroma that was effectively treated with exclusive doxycycline sclerotherapy.

## 1. Introduction

Cystic hygroma, a cystic subtype of lymphangioma, is a congenital benign lymphatic malformation. It is considered a relatively rare lesion having an incidence of 1 in 2000–4000 live births [1]. Though not fully understood, cystic lymphangiomas are thought to arise from errors with signaling during embryogenesis of the lymphatic system, resulting in abnormal lymphatic channels and cystic spaces of various sizes [2]. Cystic lymphangiomas can manifest at any age, with 50% of the cases present at birth and 90% by the end of infancy [3]. Approximately 80% of cystic hygromas occur in the cervicofacial region although they can manifest throughout the body [4].

Several classification systems have been proposed to aid in diagnosing and managing cystic hygromas. Smith and colleagues described the cystic lymphangiomas as either macrocystic, microcystic, or mixed, depending on radiological findings. Macrocysts were defined as lesions with cystic contents of at least 2 cm^3^ in volume, whereas microcysts contained lesions less than 2 cm^3^. Furthermore, this classification is also capable of predicting the response to sclerotherapy [5]. de Serres et al. also proposed a classification system based on the anatomic location of the lymphatic malformation in relation to the hyoid. This allowed for prognostication and estimation of the surgical complication rate [6].

Historically, surgery has been the cornerstone of treatment, but the total excision of the lymphangioma can lead to severe complications such as neurovascular injury and cosmetic deformity. Recent evidence has shown that macrocytic lymphangiomas treated with surgical excision also respond very well to percutaneous sclerotherapy with less surgical morbidity [7, 8]. The case report in this paper presents an 18-month-old male with a large cervical cystic hygroma which was successfully treated with doxycycline sclerotherapy by the Otolaryngology Department at Eric Williams Medical Sciences Complex (EWMSC), Trinidad and Tobago. This is the first case report of doxycycline sclerotherapy of a cystic hygroma from the English-speaking Caribbean.

## 2. Case Report

An 18-month-old male was referred to the Department of Otolaryngology by his pediatrician for an asymptomatic, aesthetically unappealing swelling. The patient's mother provided an antenatal history of him being born full term via cesarean section and subsequently noticed a small yet visible left-sided neck mass. She was advised to follow-up in the ENT outpatient clinic but defaulted from any further care. A history of an upper respiratory tract infection and progressive left-sided neck swelling that began one month prior was given.

**Figure 1:**
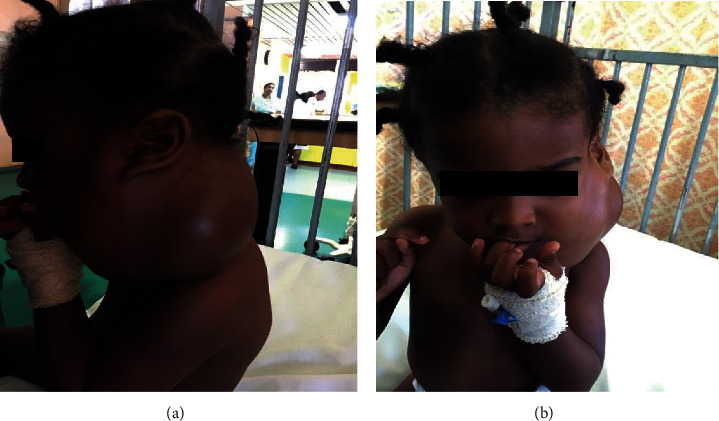
Preoperative clinical photos of left cervical cystic hygroma.

On clinical examination, the infant was in no respiratory distress. A 9 × 9 cm nontender cervical neck mass involving both the left anterior and posterior triangles was evident (Figures 1(a) and 1(b)). The neck mass was smooth, mildly compressible, and not attached to the skin. There were neither palpable lymph nodes in the neck nor any other swellings elsewhere. Intraoral examination and flexible nasolaryngoscopy was unremarkable.

**Figure 2:**
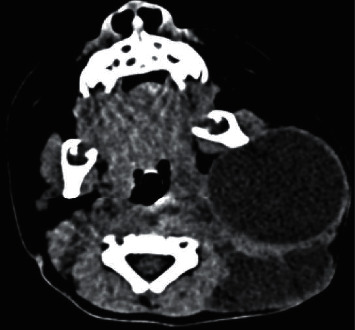
Axial computed tomography showing left cervical cystic hygroma.

**Figure 3:**
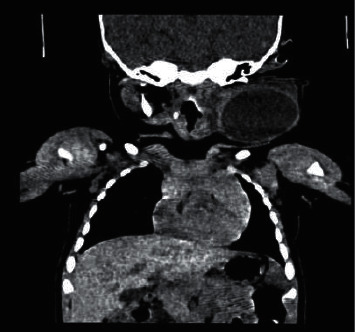
Coronal computed tomography showing left cervical cystic hygroma.

An ultrasound of the neck demonstrated two distinct complex cystic avascular masses with multiple separations and internal echoes. One mass was located anterior to the external auditory meatus measuring 3.8 × 3.4 × 3.5 cm, and the other mass was located at the left lateral cervical region measuring 7.7 × 3.4 × 7.7 cm. Based on these dimensions of the mass, a diagnosis of a left cervical cystic hygroma was made, and a computed tomography (CT) scan was arranged to acquire more precise radiological details of the mass. CT findings showed a large 9 × 5 × 9 cm well-defined left-sided neck mass extending from the subcutaneous tissues deep to the left parapharyngeal space. The lesion encased the left sternocleidomastoid muscle and contained thin internal septations with a multiloculated appearance. The medial margin of the left lesions abutted the left carotid sheath with mild displacement and compression of the left internal jugular vein. At the level of the glottis, there was encasement of the carotid sheath with a small extension to the region of the left aryepiglottic fold, extending superiorly to the left oropharyngeal region. Superiorly, the lesion extended into the left parotid gland and subcutaneous tissue posterior to the external ear (Figures 2 and 3). According to the staging system proposed by de Serres, the lesion was deemed a macrocytic, stage 3 lesion. Following an extensive discussion with the patient's parents, the decision was made to perform sclerosis of the cystic hygroma.

**Figure 4:**
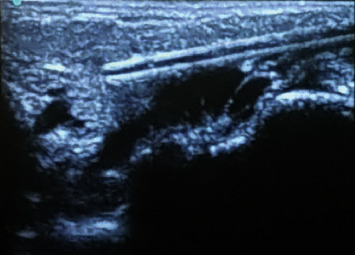
Cannulation of the cystic lesions using ultrasound guidance.

**Figure 5:**
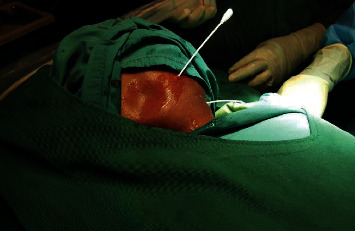
Complete aspiration of the cystic contents of the lesion.

The procedure was performed at an operating theatre under general anesthesia. The patient was cleaned and draped. Prophylactic antibiotics were given before the procedure and continued for approximately one week. Using ultrasound guidance, the macrocysts were cannulated with a 5 French Yueh Centesis catheter needle (Figure 4). The fluid contents were aspirated as thoroughly as possible, and a sample was sent for both microbiological and cytological analysis confirming a sterile cystic hygroma (Figure 5).

**Figure 6:**
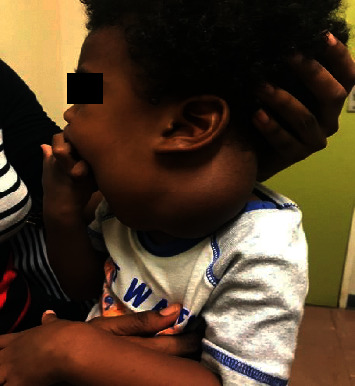
Cystic hygroma 1 month after treatment.

**Figure 7:**
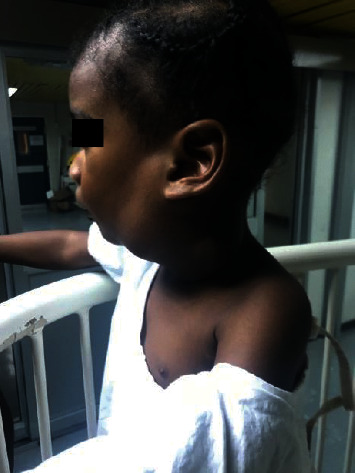
Cystic hygroma 3 months after treatment.

**Figure 8:**
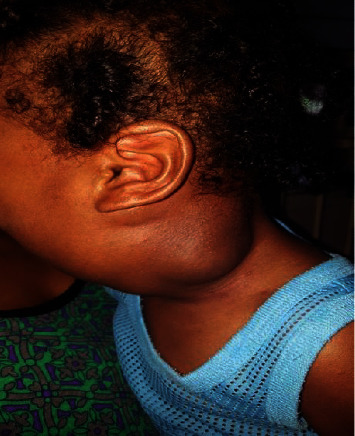
Cystic hygroma 6 months after treatment.

**Figure 9:**
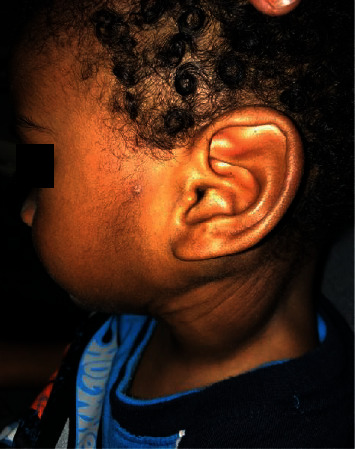
Cystic hygroma 9 months after treatment.

**Figure 10:**
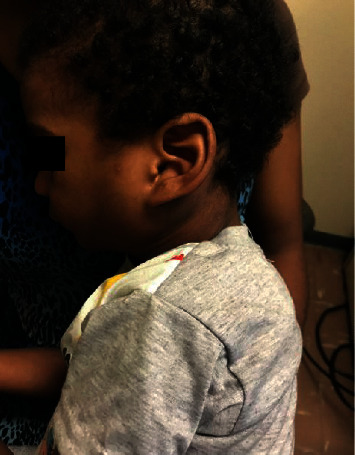
Cystic hygroma 12 months after treatment.

One hundred milliliters of 10 mg/ml doxycycline solution was reconstituted in sterile water using 100 mg doxycycline capsules. Five milliliters of Ultravist TM 300 was injected to outline the cysts fluoroscopically. The doxycycline solution was then injected into the lesion. The patient was left intubated and transferred to the Pediatric Intensive Care Unit, where he was kept sedated. The doxycycline was retained in the lesion for six hours and then actively drained. Instillation and drainage of the doxycycline solution was repeated the following day, and the catheters were removed. Subsequently, the patient was extubated and sent to the ward for 24 hours of observation and pain control. The entire procedure was repeated twice, 3 months apart (Figures 6–9). Complete resolution of the cystic hygroma was observed one year after the initial procedure (Figure 10). When examined at the outpatient clinic at 18 months, there was no recurrence or complications detected.

## 3. Discussion

Cystic lymphangiomas of the head and neck in children are often intimately involved with tissue planes, namely, the critical aerodigestive and neurovascular structures. Though several treatment algorithms for cystic hygromas have been proposed, the ideal model for management has yet to be revealed. Treatment options include watchful waiting, sclerotherapy, or surgical resection. The clinical presentation of cystic lymphangiomas is determined by their anatomic location, size, and the physiological impairment they cause. Sudden expansion may result from infections or spontaneous intralesional hemorrhage. Lesions of the cervicofacial region may not only cause airway compromise, dysphagia, elocution, and facial disfigurement but also be a source of substantial social and psychological concern to both the patient and parents [1, 7, 9].

Despite many inherent dangers associated with surgical excision, it remains the most popular method of treatment in many developing countries [1, 3]. Surgery may be numerous, intricate, and may have to be executed in strategic stages before complete excision can be achieved. Due to the infiltrative nature of cystic lymphangiomas, complete surgical excision in some instances are not only challenging but also unlikely [1]. Incomplete excision and resultant persistence of disease can often lead to recurrence of the cystic lymphangioma. The literature has highlighted recurrence rates as high as 12% to 53% in such cases [7, 10]. The conceivable complications of surgical extirpation of cystic hygroma are numerous. Intraoperatively, damage to the facial, hypoglossal and spinal accessory nerves, the carotid and internal jugular vessels, pleura, as well as the thoracic duct can occur. Post operatively, there may be evidence of wound infections, hemorrhage, seroma, and lymphatic leakage from the wound and hypertrophic scarring [4, 9, 11].

Over the past 30 years, sclerotherapy have surfaced as a favorable alternative for the treatment of cystic lymphangiomas in children in an effort to avoid the precarious complications of surgery [3, 12]. Commonly used agents include bleomycin, ethanol, doxycycline, and OK432 [11, 13]. Sclerotherapy has numerous advantages over surgical excision. Sclerotherapy requires no incisions and manipulation of overlying skin and soft tissue, and thus the cosmetic deformity is less common; in addition, the probability of nerve injury and infections are low. On the other hand, the delayed effect of sclerotherapy and the need for multiple procedures may be considered disadvantages to this treatment modality. Moreover, patients with exclusively microcystic lymphangiomas may not benefit from sclerotherapy [14]. Though each sclerosant has its individual positives and negatives, the choice of doxycycline for the index case was based on its availability and safety profile.

Doxycycline is a fairly economical and readily accessible tetracycline antibiotic with an established safety profile. Historically used for the pleurodesis of malignant effusions, its effectiveness as a sclerosant for cystic lymphangiomas was first reported by Molitch et al. [15, 16]. Since then, several case studies have demonstrated its success. Burrows et al. treated 41 patients with cystic lymphangiomas with doxycycline sclerotherapy and accomplished a response rate of approximately 83% [14]. The precise mechanism of action of doxycycline is uncertain; however, it is believed that doxycycline evokes an inflammatory reaction within the thin-walled cystic lymphangioma, and the resultant effect is involution of the cyst by deposition of collagen and fibrin. In addition, doxycycline suppresses vascular endothelial growth factor-induced angiogenesis and lymphangiogensis and inhibits matrix metalloproteinases, a key component for cell proliferation [3, 16].

There is no standard technique for doxycycline sclerotherapy, and as a result, the index sclerosis procedure was generally adopted from Molitch et al. [15]. Their method entails 3 days of consecutive sclerotherapy 24 hours apart until passive drainage from the lymphatic malformation was minimal. The duration of their technique occasionally extended to over a week. The Pediatric Intensive Care Unit (PICU) at the Eric Williams Medical Sciences Complex is limited to 5 beds as such space is critical. Based on the concept of doxycycline's mode of action, two injections of doxycycline 24 hours apart seemed sufficient to incite an inflammatory reaction on the endothelial surface of the cystic hygroma, effectively utilizing the resources and space at the institution.

Doxycycline is known to cause severe discomfort during injection and postprocedure for approximately 3 hours as the index case was done under general anesthesia. The child was kept intubated and sedated in the PICU in order to seamlessly repeat the process the subsequent day, avoid dislodgement of the percutaneous catheters, rovide adequate analgesics, and closely monitor our novel therapeutic endeavor. Relatively large volumes of doxycycline can be safely used for sclerosis without toxicity as it can be used to treat large cystic lymphangiomas [14]. As demonstrated in this case, we safely used 1000 mg of doxycycline for the initial sclerosis. Although the use of tetracyclines is generally avoided in children because of its calcium-binding effect and potential dental staining, these effects are minuscule with doxycycline [8, 14, 16].

## 4. Conclusion

Our preliminary results with doxycycline sclerotherapy as an alternative form of treatment for cervical cystic hygromas are encouraging. This case report highlights the possibility of safely achieving favorable results with doxycycline sclerotherapy in a developing country. Adequate diagnostic workup, classification and staging of cystic lymphangiomas are paramount for proper individualized treatment selection. The traditional absolute surgical option employed by developing countries need to be revised as the astounding recurrence rates and potential surgical morbidity can be avoided in some patients.

## Data Availability

No data were used to support this study.
